# Supplementary material for: Progressing Ultragreen, Energy-Efficient Biobased Depolymerization of Poly(ethylene terephthalate) via Microwave-Assisted Green Deep Eutectic Solvent and Enzymatic Treatment
Source: Polymers (Basel). 2021 Dec 29;14(1):109. doi: 10.3390/polym14010109 (PMC8747168; doi:10.3390/polym14010109)
Supplement: Supplementary file 1 [file polymers-14-00109-s001.zip › polymers-1485587-supplementary.pdf]

Supporting Information

Progressing Ultragreen, Energy-Efficient Biobased Depolymerization of Poly(Ethylene Terephthalate) via Microwave-Assisted Green Deep Eutectic Solvent and Enzymatic Treatment

Olivia A. Attallah [1,2,†], Muhammad Azeem [1,*,†], Efstratios Nikolaivits [3], Evangelos Topakas [3] and Margaret Brennan Fournet [1]

[1] Materials Research Institute, Technological University of the Shannon: Midlands Midwest,
N37 HD68 Athlone, Ireland; oadly@ait.ie (O.A.A.); mfournet@ait.ie (M.B.F.)

[2] Pharmaceutical Chemistry Department, Faculty of Pharmacy, Heliopolis University, Cairo-Belbeis Desert Road, El Salam, Cairo 11777, Egypt

[3] Industrial Biotechnology & Biocatalysis Group, Biotechnology Laboratory, School of Chemical Engineering, National Technical University of Athens, Athens 15780, Greece; stratosnikolai@gmail.com (E.N.);
vtopakas@chemeng.ntua.gr (E.T.)

* Correspondence: m.azeem@research.ait.ie

† These authors contributed equally to this work.

Note:

Number of Pages 5

Number of Figures S1

Number of Tables S1

Table of Content

Table S1: Different compositions of DESs used in PET recycling 3

Figure S1: Post-enzymatic hydrolysis products 4

TableS1. *Different compositions of DESs used in PET recycling*

**DES composition**			**Reaction conditions**			**Results**	**Reference**
HBA	HBD	Ratio (HBA:HBD)	Reaction time	Depolymerising agent	Temperature		
Choline chloride	Glycerol	1:2	80-100 s	Glycerol, NaOH	-	38-61% weight loss of PET	[1]
Choline chloride	Zinc chloride	1:1–1:3	30 min	Diethanolamine, ethanolamine	-	THETA, TPA, BHETA (82, 83 and 95 yield%)	[2]
	Urea	1:2					
Potassium carbonate	Ethylene glycol,	1:6	2 h	ethylene glycol	180 °C	BHET (88 yield%)	[3]
Choline chloride	Urea, Dimethyl urea, Ethylene glycol, Glycerol	1:2					
Zinc chloride, zinc acetate, manganese acetate	Urea	1:12 to 1:6	30 min	ethylene glycol	170 °C	bis(hydroxyalkyl) terephthalate (83 yield%)	[4]
1-methyl-3-butylimidazolium chloride	Zinc chloride, Manganese chloride	1:1	5.0 h	ethylene glycol	190 °C	BHET (83.8 yield%)	[5]
Choline chloride	Ethylene glycol	1:2	120 s	ethylene glycol, NaOH	144 °C	45.8% weight loss of PET	[6]

Hydrogen bond donor (HBD); hydrogen bond acceptor (HBA); (2-hydroxyethyl)-terephthalamide (THETA); Terephthalic acid: (TPA); bis (2-hydroxy ethylene) terephthalamide: (BHETA); bis(hydroxyethyl) terephthalate (BHET); sodium hydroxide (NaOH)

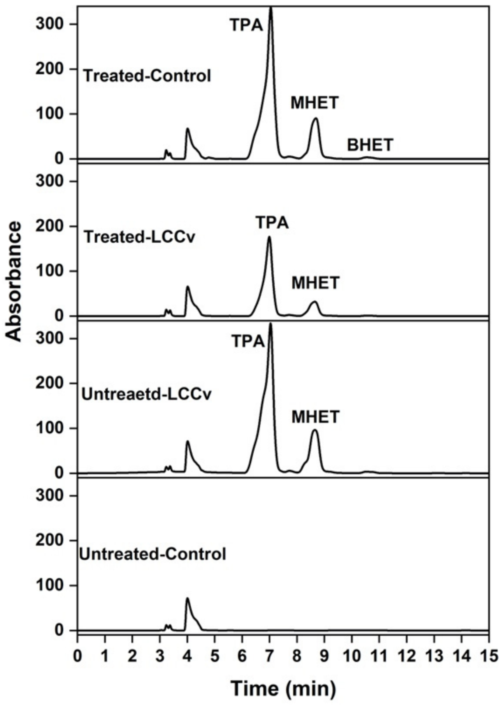

Figure S1. HPLC chromatograms of post-enzymatic hydrolysis products

[1] S. Choi and H. M. Choi, “Eco-friendly, Expeditious Depolymerization of PET in the Blend Fabrics by Using a Bio-based Deep Eutectic Solvent under Microwave Irradiation for Composition Identification,” Fibers Polym., vol. 20, no. 4, pp. 752–759, 2019.

[2] R. M. Musale and S. R. Shukla, “Deep eutectic solvent as effective catalyst for aminolysis of polyethylene terephthalate (PET) waste,” Int. J. Plast. Technol., vol. 20, no. 1, pp. 106–120, 2016.

[3] E. Sert, E. Yılmaz, and F. S. Atalay, “Chemical Recycling of Polyethlylene Terephthalate by Glycolysis Using Deep Eutectic Solvents,” J. Polym. Environ., vol. 27, no. 12, pp. 2956–2962, 2019.

[4] Q. Wang, X. Yao, Y. Geng, Q. Zhou, X. Lu, and S. Zhang, “Deep eutectic solvents as highly active catalysts for the fast and mild glycolysis of poly(ethylene terephthalate)(PET),” Green Chem., vol. 17, no. 4, pp. 2473–2479, 2015.

[5] Q. F. Yue, L. F. Xiao, M. L. Zhang, and X. F. Bai, “The glycolysis of poly(ethylene terephthalate) waste: Lewis acidic ionic liquids as high efficient catalysts,” Polymers (Basel)., vol. 5, no. 4, pp. 1258–1271, 2013.

[6] J. Y. Cho, H. M. Choi, and K. W. Oh, “Rapid hydrophilic modification of poly(ethylene terephthalate) surface by using deep eutectic solvent and microwave irradiation,” Text. Res. J., vol. 86, no. 12, pp. 1318–1327, 2016.
